# Ductility performance of reinforced rubberised concrete beams incorporating burnt clay powder

**DOI:** 10.1016/j.heliyon.2021.e08310

**Published:** 2021-11-02

**Authors:** David Sinkhonde, Richard Ocharo Onchiri, Walter Odhiambo Oyawa, John Nyiro Mwero

**Affiliations:** a Department of Civil and Construction Engineering, Pan African University Institute for Basic Sciences, Technology and Innovation, Nairobi, Kenya; b Department of Building and Civil Engineering, Technical University of Mombasa, Mombasa, Kenya; c Department of Civil, Construction and Environmental Engineering, Jomo Kenyatta University of Agriculture and Technology, Nairobi, Kenya; d Department of Civil and Construction Engineering, University of Nairobi, Nairobi, Kenya

**Keywords:** Burnt clay brick powder, Ductility, Rubberised concrete, Shear, Flexure, Seismic application

## Abstract

Application of rubberised concrete in earthquake prone areas is of significant importance. Although investigations have been conducted to research on the ductility of rubberised concrete, the behaviour of rubberised concrete with Burnt Clay Brick Powder (BCBP) is not well understood. This paper captures the ductility behaviour of rubberised concrete containing BCBP. In this study, 3 beams were investigated in flexure while the other 3 beams were made to fail in shear and flexure. For the beams that failed in flexure, ductility of concrete beams containing 5% BCBP and 10% Waste Tire Rubber (WTR) improved by 23.47% compared to control beam. This increase in ductility was evidenced with only 15.31% reduction in flexural load. Moreover, the beam containing 5% BCBP and 10% WTR failing in shear and flexure exhibited 14.59% ductility improvement with 16.33% load reduction in comparison to the control beam. Eventually, the study concluded that it is possible to achieve improved ductility without substantial loss in ultimate failure load by using 5% BCBP and 10% WTR. Such properties demonstrated that this rubberised concrete with 5% BCBP can be used in seismic applications.

## 1 Introduction

Majority of used tires are damped and stockpiled in landfills. For instance, nearly 1000 million tires conclude their life each year of which more than 50% are disposed without any treatment [1]. Waste Tire Rubber (WTR) is a main non-biodegradable waste which is hazardous to the surrounding [2, 3]. The use of WTR in concrete production has been reported to significantly assist in recycling of tires generated across the world [4]. Earthquakes are increasingly becoming devastating in recent times as a result of the ever increasing population and urbanization [5]. The inclusion of WTR substituting fine or coarse aggregates in concrete advances the ductility properties of concrete [6, 7, 8]. This property is desired for structural members since it accommodates distribution of stresses and offers warning about an anticipated failure [9]. Ductility property is also significant in high rise buildings which are prone to earthquake failures. Structural engineers have a great role to play in responding to the consequences arising from earthquakes [10].

Displacement ductility is measured using ductility index from load-deflection graphs. This index is the ratio of deflection at ultimate load to deflection at yield point of steel [11]. Other studies [12], investigated on replacement of coarse aggregate with tire rubber. It was revealed that increase in coarse aggregates substitution with WTR up to 25% enhanced curvature ductility. Strain capacity accompanied by tire rubber incorporation resulted in increased ductility. Meanwhile, seismic behaviour of structures has been found to significantly improve due to increased energy dissipation illustrated by tire rubber inclusion [13]. In another study [14], increasing tire rubber content from 20% to 50% limited the capability of beams to undergo high deformation and ductility. This behaviour was attributed to weak bonding illustrated by tire rubber content in concrete. The presence of tire rubber in cement-based composites has also been observed to enhance ductility irrespective of the size elsewhere [15].

The use of pozzolanic materials in concrete production is becoming an essential approach towards mitigating environmental concerns due to cement production [16, 17]. Stockpilings of waste clay bricks occupy significant land especially for cities without sufficient dumping sites [17]. The use of BCBP in concrete beneficially contributes to environmental sustainability in two folds: lowering demand for Portland cement and wise alleviation of fragmented bricks from the environment [17, 18]. Previous investigations on use of BCBP have mainly focused on concrete cubes, cylinders and unreinforced beams with limited studies on reinforced concrete beams.

This research investigated the influence of WTR on flexure, shear, ductility and cracking behaviour of reinforced concrete beams especially with addition of BCBP. The construction industry should consider the improvement of ductility properties of reinforced beams while achieving sustainability in construction. The inclusion of WTR and BCBP could present potential enhancement of ductility performance of concrete beams apart from reducing environmental pollution. This sustainable construction is also an economically sound construction method.

## 2 Materials and methods

### 2.1 Materials properties

**Figure 1:**
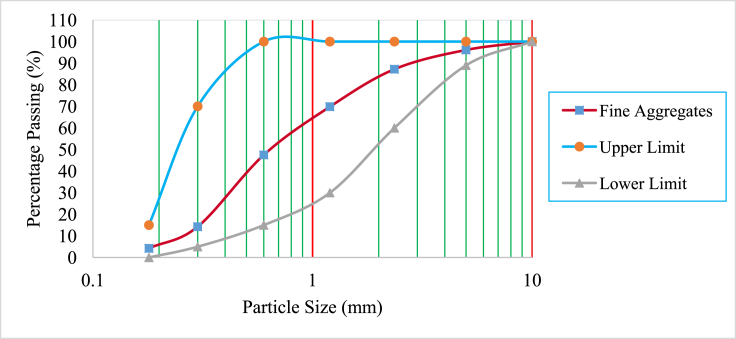
Grading curves of fine aggregate.

**Figure 2:**
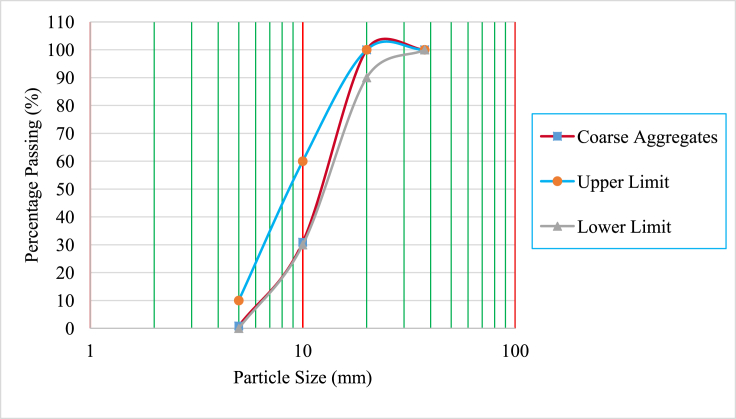
Grading curves of coarse aggregate.

**Figure 3:**
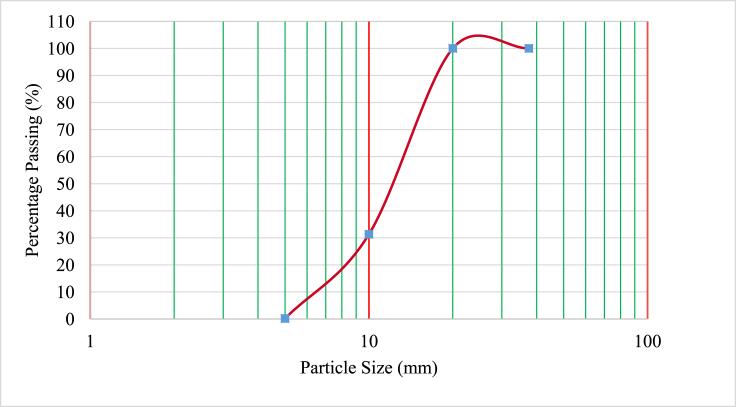
Grading curve of tire rubber.

Ordinary Portland Cement (OPC) class 42.5 meeting specifications in the standard [19], was used. This OPC showed acceptable specific gravity and Le Chatelier soundness values of 3.12 and 5.7 mm respectively. BCBP of specific gravity 2.69 and passing through 0.075 mm sieve was used in this study. The chemical compositions of cement and BCBP are tabulated in Table 1. As shown in the table, the sum of (SiO_2_ + Fe_2_O_3_ + Al_2_O_3_) of greater than 70% in BCBP conformed to the requirements in the code [20], as Class N pozzolan used as cementing material in concrete. BCBP was then used to partially replace cement during concrete production. Crushed stone aggregate with maximum size 20 mm and natural sand were used as coarse aggregates and fine aggregates respectively. Fine aggregates had specific gravity of 2.59 and water absorption of 1.11% whereas coarse aggregates had specific gravity of 2.45 and water absorption of 3.39%. WTR of maximum size 20 mm had specific gravity and water absorption values of 1.14 and 0.98% respectively. In this study, WTR was used to partially replace coarse aggregate in concrete. The gradations of fine aggregates, coarse aggregates and tire rubber are presented in Figures 1, 2, and 3 respectively. Steel bar diameters of 8 mm and 12 mm with average yield strengths of 540.74 MPa and 492.50 MPa respectively, were used for the tested reinforced concrete beams.

**Table 1:** The chemical compositions of cement and BCBP.

Material	SiO_2_	Fe_2_O_3_	Al_2_O_3_	CaO	MgO	Na_2_O	K_2_O	TiO_2_	MnO	P_2_O_5_	Ba	S	LOI
Cement	15.45	4.55	2.81	62.45	-	0.48	1.01	0.47	0.12	1.29	0.05	2.75	7.47
BCBP	64.36	12.86	8.71	2.00	-	1.82	3.05	2.13	0.68	1.18	1.18	-	0.97

### 2.2 Concrete mixtures and tests for fresh and hardened concrete

Class 30 concrete mix in accordance to British Research Environment (BRE) was selected for the beams. The mix design procedure has been presented in another study [21]. Before adopting the mix design for class 30, preliminary trial mixes were conducted. The findings revealed that water-cement ratio of at least 0.45 was to be used to obtain fresh concrete with slump values from 10 to 30 mm. Three investigated mixes were coded 0P0T (control concrete), 5P0T (mix with 5% BCBP) and 5P10T (mix with 5% BCBP and 10% WTR). The compressive strength tests were conducted on 100 × 100 × 100 mm cubes exposed to the same curing period as that of beams in accordance with the code [22]. Slump tests were performed on fresh concrete with a slump cone in conformance with the standard [23]. The standard gives the instrument dimensions as 300 mm for height, 100 mm for top diameter, 200 mm for bottom diameter and metal thickness of 1.60 mm.

### 2.3 Flexure, shear and ductility tests of reinforced beams

#### 2.3.1 Specimen preparation, instrumentation and casting

The flexural, shear and ductility properties of rubberised concrete containing BCBP were assessed by testing 9 beams of 150 × 200 × 2000 mm dimensions in accordance with the code [24]. The design of beams was done for Class 30 concrete in conformity with the standard [25]. The beams for flexure were coded B1/0P0T, B2/5P0T and B3/5P10T similar to codes used for concrete cubes. Beams for shear and flexure were coded B4/0P0T, B5/5P0T and B6/5P10T. Stiff timber moulds were prepared to restrain any movements during concrete placement. To account for steel slippage from concrete, the ends of longitudinal reinforcement bars were hooked. All scheduling, dimensioning and bending of steel reinforcement were carried out in accordance with the code [26]. Before casting, installation of electrical résistance strain gauges (type PFL-10-11-3LJC-F and gauge resistance 118 ± 0.5 Ω supplied by Tokyo Sokki Kenkyujo Co. Ltd, Japan) and lubricating moulds were conducted for strain measurements and facilitating demoulding respectively.

To facilitate strain gauge fixing, the tension bars at mid-span were smoothened using a bench grinder. The strain gauges were carefully embedded on steel bars using glue followed by covering with water proof material for protection. Steel reinforcement bars were transferred in the timber moulds. While keeping the moulds horizontally, the casting of the samples into the moulds was performed. Correspondingly, several cubes were cast to establish the compressive strength properties of concrete. This was followed by compaction which used a poker vibrator. After 24 h, the specimens were de-moulded without damage to either concrete or the moulds. They were then moist-cured by covering using wet gunny bags which ensured uniform curing. The samples were kept inside the laboratory having humidified air and room temperature for 28 days.

#### 2.3.2 Testing of the beam

**Figure 4:**
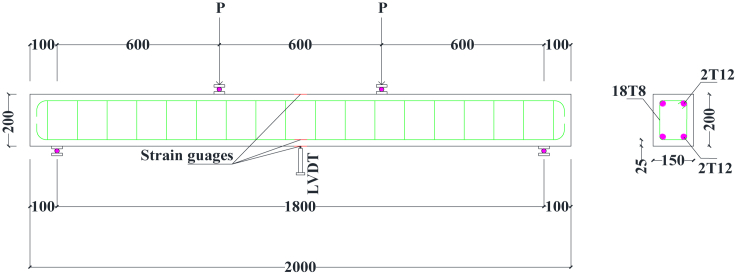
Testing beams in flexure.

**Figure 5:**
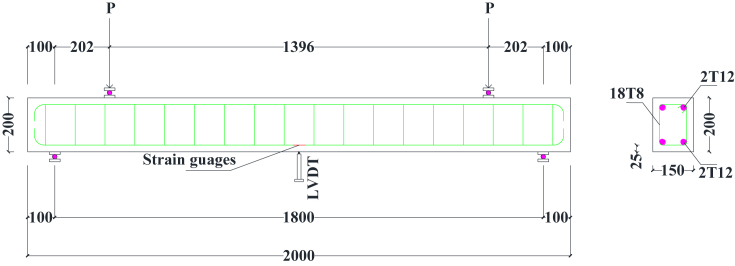
Testing beams in shear and flexure.

The specimen for flexural testing was mounted on rollers which provided supports as schematically illustrated in Figure 4. A Linear Variable Differential Transducer (LVDT) was instrumented at the mid-span of beam soffit for mid-span deflection detections. During test initiation, loading positioning was at the centre of the beam and perpendicular to the specimen face. The load from a hydraulic jack of 400 kN capacity was then continuously applied at constant pressure until maximum load leading to failure was attained. A 200 kN capacity load cell was used to measure the applied load. Deflections were observed using LVDT connected to a data logger for each load increment. The maximum load was recorded and the flexural strength was computed using Eq. (1). Load-deflection curves were plotted to establish the ductility of the specimen. The beams were continuously inspected to obtain details of crack developments. After the test was terminated, inspection of beams continued to establish crack spacing, crack patterns and failure modes. The other beams were tested by placing loads at 202 mm from the supports (Figure 5). Computations achieving this distance were done using shear reinforcement details provided during beam design for flexure. The aim of positioning the loads at this distance from the supports was to enhance the possibility of shear and bending failure. There were 9 beams in total, 3 were used to investigate flexural strength, 3 were repeat and 3 were used to investigate shear and bending failure.

(1) $${\text{f}}_{\text{cf}}=\frac{3FI\;}{2bd^{2}}$$

According to Eq. (1), f_cf_ is the flexural strength in megapascal (MPa), F is the maximum load in Newton (N), b is the thickness of the specimen in mm and d is the depth of the specimen in mm.

## 3 Results and discussion

Several aspects relating to beam failures such as flexural, shear, ductility and cracking behaviour are discussed in two parts. The first part (Section 3.1) focuses on beams that failed in flexure. The second part (Section 3.2) discusses findings obtained after upgrading the setup to study the performance of beams in shear and bending.

### 3.1 Results of beams tested for flexure

#### 3.1.1 Compressive strength

**Figure 6:**
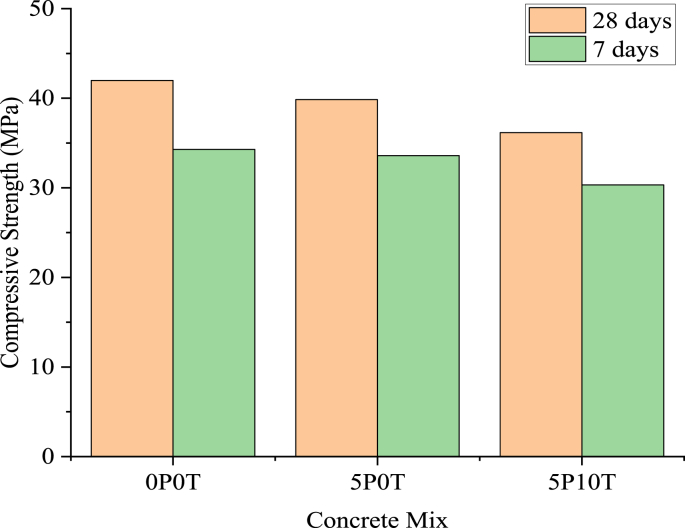
Compressive strength results of developed mixtures.

Figure 6 represents 7 and 28-days compressive strength results for the tested mixes. The introduction of 5% BCBP resulted in compressive strength losses at both 7 and 28 days. At 28 days, B2/5P0T showed a reduction in strength of 5.1% compared to the control beam. Increased strength losses were noticed when tire rubber aggregates were introduced. Replacement of coarse aggregates by 10% rubber resulted to a 13.87% loss of compressive strength in comparison with control concrete.

The findings of compressive strength were used to verify the characteristic strength of concrete used during casting beams. It is clear from Figure 6 that the characteristic strength of 30 MPa was exceeded in all mixes after 28 days curing period. The decrease in compressive strength of concrete with 5% BCBP is an illustration that concrete cannot fully benefit from the pozzolanic reaction of BCBP at early curing periods. BCBP requires longer curing periods to actively participate in pozzolanic reaction [27]. Meanwhile, the decrease in strength due to the inclusion of tire rubber suggests the existence of high compressibility of tire rubber and weak bonding between tire rubber and surrounding concrete constituents [28, 29]. An additional probable reason can be the initiation of air content accompanied by the inclusion of tire rubber [30]. This increase in air content negatively affects concrete mechanical properties [30].

#### 3.1.2 First crack load, ultimate load and mid-span deflections

**Figure 7:**
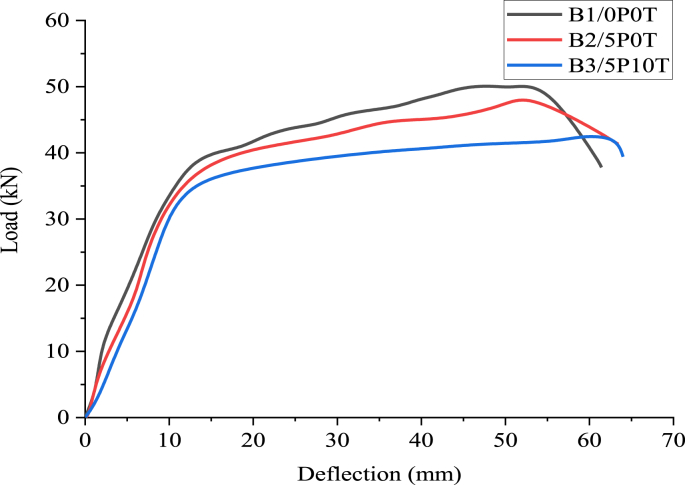
Experimental load-mid-span deflection responses.

Findings on loads and the number of cracks for each beam can be found in Table 2. Figure 7 shows the load-mid-span deflection responses of the investigated beams. In this Figure 7, the linearity is seen up to the yield point of longitudinal steel. A significant deviation of the slope is then observed after the yielding of steel. During visual inspection of the beams, the first cracking loads were between 15 kN and 23 kN for the tested beams (Table 2). Further increase of load led to concrete crushing in the compression zone and overall beam failure. The failure loads of the collapsed beams ranged from 40 kN to 51 kN.

**Table 2:** Results of the flexural test.

Beam ID	First Crack Load (kN)	First Crack Deflection (mm)	Failure Type	Number of Cracks
B1/0P0T	23	6.234	Flexure	7
B2/5P0T	18.4	6.281	Flexure	6
B3/5P10T	16.8	6.4	Flexure	13

As expected, the reduction of the first crack load and the ultimate load was observed when cement was replaced by 5% BCBP. This might be due to the limited curing period. Visual observation of beam failure showed that the introduction of tire rubber resulted in a reduction of first crack load. The suggested reason for this behaviour is the significant reduction of flexural strength of rubberised concrete [30]. Weak bonding between tire rubber and other concrete ingredients accounts for this reduction [7]. Therefore, this reduction reflects that rubberised concrete containing BCBP achieves the first crack at a lower load compared to normal concrete. As the loading progressed, the flexural cracks were extended and new cracks propagated away from the mid-span. During the loading process, the slope of the load-deflection curve (flexural stiffness) reduced due to the inclusion of tire rubber. The flexural stiffness values of 3.352 and 2.943 were computed for control concrete and rubberised concrete containing BCBP respectively. A decrease in modulus of elasticity of rubberised concrete was suggested as a cause of such behaviour [4].

From Figure 7, the first crack deflection of rubberised concrete containing BCBP is 2.663% greater than for normal concrete. A lower load of 16.8 kN corresponded to this deflection. The ultimate deflection of rubberised concrete with 5% BCBP increased by 12.01% compared to control concrete. Such an increase in deflection is an indicator of strain capacity [31]. The enhancement in deflection is likely due to improved energy dissipation attributed to the reduced modulus of elasticity of rubber [32]. Also, under similar loading circumstances, steel reinforcement bars in structural rubberised concrete exhibit large strains compared to normal concrete [4].

#### 3.1.3 Concrete strain

**Figure 8:**
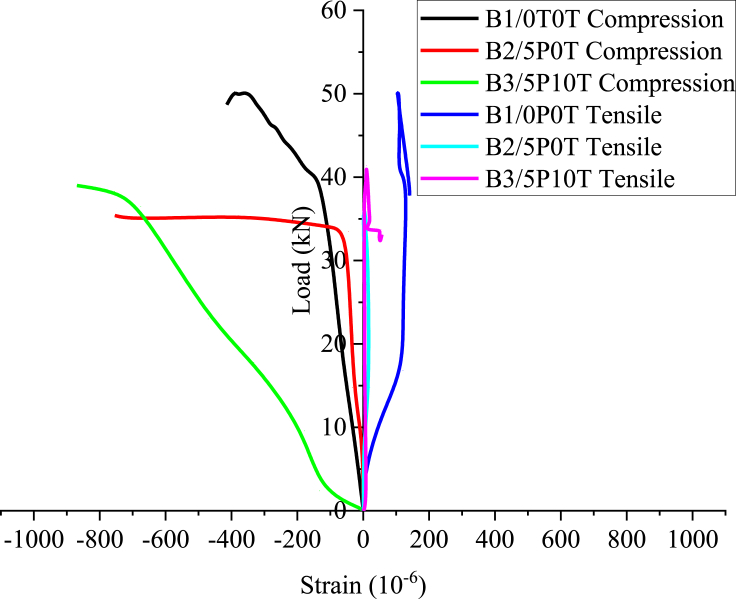
The experimental load-strain curves for mid-points of top and bottom concrete surfaces.

Load-strain curves for mid-points of top and bottom concrete surfaces have been presented in Figure 8. The negative strains and positive strains in the figure represent compressive and tensile strains respectively. The maximum compressive strains were -837.32 × 10^−6^, -938.755 × 10^−6^ and 960.765 × 10^−6^ for B1/0P0T, B2/5P0T and B3/5P10T respectively. The tensile zone gave maximum strains of 155.024 × 10^−6^, 20.096 × 10^−6^ and 59.33 × 10^−6^ for B4/0P0T, B5/5P0T and B6/5P10T respectively. These strains were monitored during the entire duration of loading using strain gauges positioned at the top and bottom surfaces of the beams. The findings generally indicate increase in strains as the loading increased. This increase was more pronounced in strain gauges embedded at the top surface.

In Figure 8, the highest negative slope of load-strain was observed for the compression zone of rubberised concrete containing BCBP. Such behaviour also proved the existence of low modulus of elasticity in rubberised concrete. Unfortunately, the strain gauges at the steel reinforcement were inoperative a few seconds after the loading commenced. Stretching of bottom concrete particles during early crack formation was speculated as a cause for that behaviour. Slipping of strain gauges during the loading process might have probably been another cause.

#### 3.1.4 Cracking behaviour

**Figure 9:**
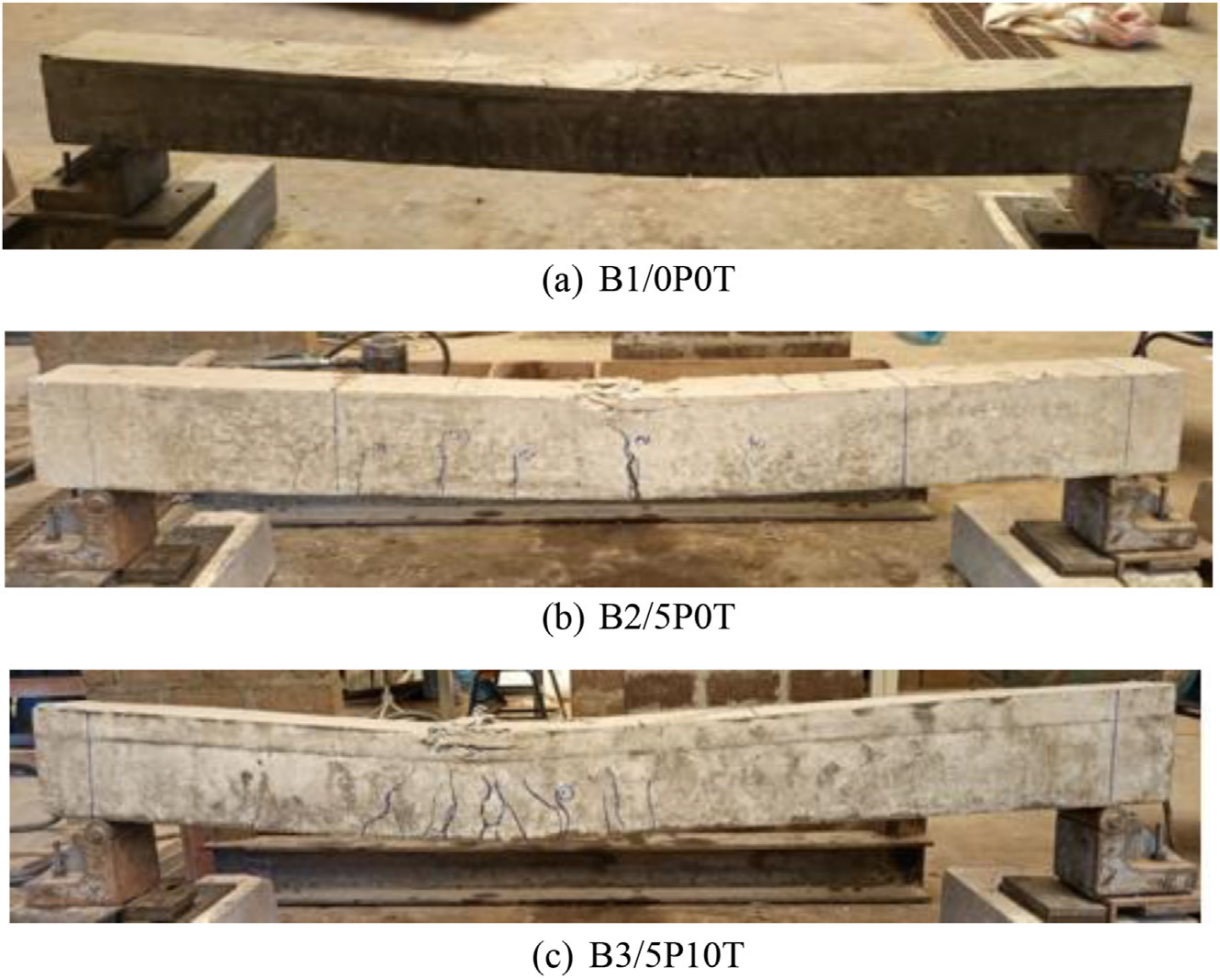
Crack patterns and failure modes of beams.

Figure 9a, b and c show the cracking pattern and failure modes of the tested beams which were observed after careful visual inspection. As observed in the figures, the cracks were mainly confined to the mid-section of the beams. This was an illustration of flexural cracks resulting from the flexural failure of the tested beams.

Crack development was enhanced in rubberised concrete with BCBP compared to the control concrete. An increase in mid-span deflection (Figure 9c) due to the inclusion of tire rubber, is reasoned to be the cause of such behaviour [30]. Zigzag cracks with branches were evidenced in rubberised concrete containing 5% BCBP (Figure 9c) while relatively straight and vertical cracks were observed in B1/0P0T and B2/5P0T concrete mixtures. Crack branching occurs when micro-cracks preceding the crack tip are arrested thereby resulting in more energy needed for the propagation of cracks [33]. Branching of cracks and redistribution of stresses accompanied by the process of failure result in the ductile performance of concrete [34]. The incorporation of tire rubber also seemed to restrain the wide opening of the cracks (Figure 9c). This is in agreement with other researchers [12], who found that conventional concrete exhibits wider cracks compared to rubberised concrete. The reduction in crack width can be attributed to a reduction in failure load behavior seen in Figure 6 due to the inclusion of tire rubber [35]. Tensile steel strains responsible for crack development are in return reduced thereby resulting in decreased crack width [36].

The number of cracks increased in rubberised concrete containing BCBP compared to normal concrete. Figures 9a and 9b indicate comparable number of cracks between B1/0P0T and B2/5P0T. The inclusion of 5% BCBP replacing cement in concrete was not believed to be sufficient enough to induce big differences in number of cracks. In contrast, rubberised concrete with 5% BCBP (Figure 9c) showed an increased number of closely spaced cracks compared to normal concrete. The existence of closely spaced cracks in large quantities is an illustration of reduced rubberised concrete with BCBP stiffness confirmed by low load deflection slope (Figure 7) [35]. This mechanism is observed to emanate from the low modulus of elasticity and high Poisson's ratio of tire rubber resulting in a considerable differential strain rate between concrete ingredients and tire rubber [35].

#### 3.1.5 Flexural and curvature ductility properties

**Figure 10:**
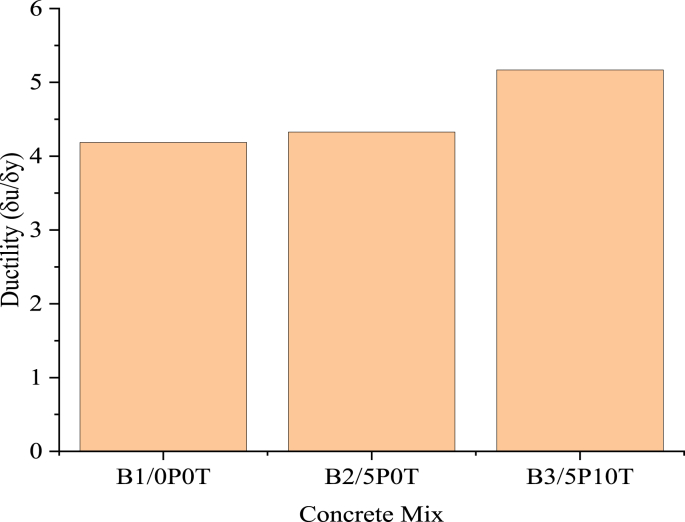
Effect of WTR content on ductility of concrete incorporated with BCBP.

Figure 10 summarises the effect of WTR content on the ductility of concrete incorporated with BCBP. Ductility is the capacity of a material to withstand an increased permanent deflection when subjected to loading up to failure [37]. It enables constant moments under deformation accompanied by plastic hinge conditions [38]. The ductility of concrete improved due to the incorporation of BCBP. A slight improvement in ductility of 3.38% was noticed in concrete incorporated with 5% BCBP. Replacing 10% of coarse aggregates with tire rubber resulted in improvement in ductility by 23.47% compared to the control beam. Deflection and ductility ratio values for each beam are given in Table 3. The table illustrates that rubberised concrete with BCBP illustrated higher ductility index compared to control concrete.

**Table 3:** Ductility index and mid-span deflection.

Beam ID	Deflection at yield (mm)	Deflection at failure (mm)	Ductility ratio
B1/0P0T	12.582	52.673	4.19
B2/5P0T	12.101	52.37	4.33
B3/5P10T	11.414	59	5.17

It is clear from Table 3 that rubberised concrete with 5% BCBP showed increased deflection at failure compared to normal concrete. The inclusion of tire rubber was found to increase the ductility of concrete (Figure 10). Previous studies point out significant reasons for such behaviour. WTR has been observed to enhance the damping behaviour of concrete in the viscoelastic vicinity [39]. The strain capability quantified from rubberised concrete demonstrates its ability to undergo many deflections before failure [31]. The resulting deflections are significant in the provision of ample warning in cases of earthquake occurrences [39]. Considering this, it is interesting to observe that rubberised concrete with BCBP can be used for structural components concerning dynamic and damping behaviour [40]. Rubberised concrete is observed to promote the seismic performance of bridges and buildings by increasing energy dissipation [39]. The authors reasoned that the requirement for the installation of mechanical dampers is reduced in instances where rubberised concrete is applied. Moreover, it is worth emphasizing that environmental aspects of concrete through the use of waste materials should be accompanied by strength and ductility considerations [41]. In this way, acceptable eco-mechanical behaviour of concrete could be achieved consequently.

#### 3.1.6 Concrete crushing

The compression zone of rubberised concrete containing BCBP heavily suffered from a large extent of damage compared to the control concrete (Figure 9c). The load-strain curves (Figure 8) illustrate the capacity of rubberised concrete to undergo large strains before failure. A larger concrete strain of 958.85 × 10^−6^ for rubberised concrete with BCBP compared to 837.32 × 10^−6^ for control concrete confirmed that tire rubber contributed to enhancing the strains. Stress concentrations from tire rubber with low stiffness are thought to lead to increased strains [42]. Also, owing to the excellent inherent deformation characteristics of rubber [43], rubberised concrete can undergo higher elastic deformation under load thereby increasing the concrete strains [42].

### 3.2 Results of beams tested for shear and bending

#### 3.2.1 Load-deflection behaviour

**Figure 11:**
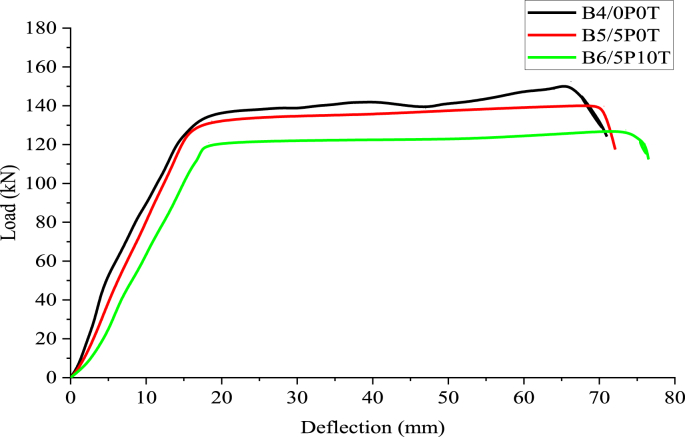
Experimental load-mid-span deflection responses.

The load plotted against the deflection graph for the tested beams (B4/0P0T, B5/5P0T and B6/5P10T) is shown in Figure 11. The curves can be generally categorised into three regions; the first region occurring up to the first crack location, the post-crack section occurring up to yield point and finally post-yield section occurring until failure. Continued deflection with a decrease in load was observed after ultimate failure for all the tested beams. Comparison of ultimate failure loads indicates that concrete containing 5% BCBP showed a 7.93% reduction of ultimate failure load compared to control concrete. A 16.33% reduction in ultimate failure load was noticed for rubberised concrete containing 5% BCBP compared to control concrete. The reduction of yield load of 10.82% was noticed for rubberised concrete containing 5% BCBP compared to control concrete. The locations of ultimate deflections for B5/5P0T and B6/5P10T are beyond that of B4/0P0T.

A closer look at Figure 11 reveals that rubberised concrete with BCBP sustained a lower load compared to control concrete. This behaviour appeared to arise because of rubber particles softness due to the reduced modulus of elasticity. In a previous study on rubberised concrete [44], the modulus of elasticity was observed to depend on the unit weight of concrete. Herein, rubberised concrete with 5% BCBP exhibited low unit weight compared to normal concrete. This might be the cause of such a reduced load. Also, the aggregate interlock contributing to 35–50% of the beam shear capacity is weakened by soft tire rubber aggregates [45]. The test results illustrate that incorporation of 5% BCBP led to a reduction of beam stiffness confirmed by a reduced load-deflection slope. This might be due to reduced beam bonding occasioning from loss of adhesion within the steel-concrete interface [46]. Satisfactory enhancement of adhesion in concrete containing BCBP can be obtained at longer concrete curing periods [27, 47]. The ultimate shear-flexure failure was demonstrated by a mixture of diagonal shear cracks and flexural cracks for all tested beams.

From Figure 11, rubberised concrete with BCBP is observed to illustrate a higher ductility ratio compared to control concrete. The increased number of cracks observed during beam inspection appeared to have predicted such behaviour. Low Poisson's ratio and modulus of elasticity of tire rubber induce differential strains between tire rubber and other concrete ingredients [30]. This phenomenon is observed to result in high tensile stress at this interface resulting in more crack propagation in comparison with the control concrete [30]. Some researchers [48], have also reported that reduced concrete compressive strength illustrated by rubberised concrete results in increased ductility.

#### 3.2.2 Failure mode and cracking behaviour

The effect of tire rubber and brick powder inclusion in concrete beams is summarised in Table 4. Also indicated in the table are the failure modes of all the tested beams. In the table, the control beam had the lowest number of cracks compared to the modified beams. Even though the number of cracks for B5/5P0T and B6/5P10T specimens was the same, the measured ultimate failure loads and deflections were different. This difference is visible in Figure 11.

**Table 4:** Summary of the results for the tested beams.

Beam Type	Beam ID	First Crack Load (kN)	Failure Type	Yield Displacement (mm)	Ultimate Displacement (mm)	Ductility Ratio	No. of Cracks
B4	0P0T	61.2	Shear- flexure	18.9	66.242	3.50	13
B5	5P0T	58.3	Shear- flexure	18.7	69	3.69	15
B6	5P10T	55.3	Shear- flexure	18.3	73.5	4.02	15

**Figure 12:**
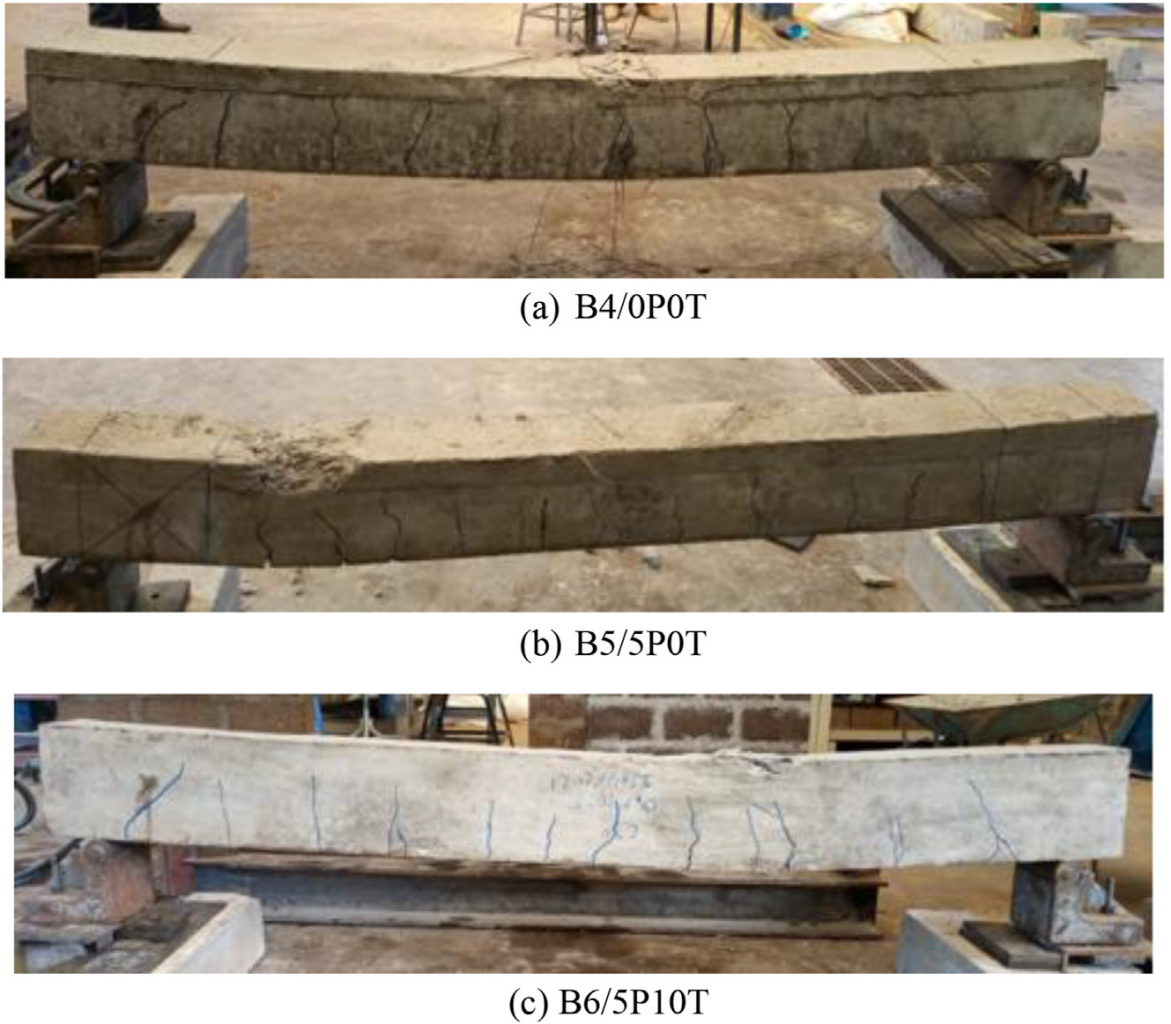
Crack patterns and failure modes of beams.

Figure 12a, b and c present the crack pattern, crack spacing and failure mode of all beams at failure. As seen from the figures, the formation of a single major crack at the supports propagating upwardly suggests a shear failure of the beams (Figure 12a, b and c). Flexural cracks positioned close to mid-span were also seen during load initiation. The formation of flexural cracks was increased farther from the mid-span after additional load application. Clearly, this showed that the failure of these beams was not limited to the mid-section only as was the case with beams that failed in flexure previously discussed. Further increment of load resulted in concrete crushing in compression vicinity and the ultimate beam failure. The crack spacing values ranged from 44 to 267 mm. The maximum crack spacing values for B4/0P0T, B5/5P0T and B6/5P10T were found to be 267 mm, 219 mm and 207 mm respectively.

The reduced number of cracks in the control beam compared to the modified beams implied that modified beams had better distribution of strains. This phenomenon was also observed in beams that failed in flexure discussed previously. Larger stiffness of the control beam was suggested to result in a reduced number of cracks. However, the existence of the same number of cracks between B5/5P0T and B6/5P10T was surprising considering the discussion about deflection. From this observation, it seemed that positioning the loads at 202 mm from supports had negligible effect on the number of cracks compared to the deflection. The lower range of 2 cracks suggested that the inclusion of BCBP and WTR did not illustrate big differences in crack number among the beams compared to the beams that failed in flexure.

The incorporation of tire rubber allowed the beams to undergo large deflections and increased the number of cracks ahead of ultimate failure. A probable justification for this behaviour is the reduction of modulus of elasticity due to the inclusion of tire rubber [35]. Such behaviour is desirable as a reduction in seismic damage can be achieved by allowing ample warning to building occupants during earthquake occurrences [49, 50]. Partially replacing coarse aggregates with tire rubber reduced the maximum cracking spacing by 22.47% compared to the control beam. These findings agreed with the results of other studies [51], where maximum crack spacing also decreased in rubberised concrete in comparison to control concrete. Crack spacing has been found to directly relate to crack width with acceptable precision [52]. The inclusion of tire rubber in concrete has been observed to contribute to crack width reduction due to enhanced energy absorption of tire rubber [30]. This behaviour is illustrated in Figure 12c. Ductile failure of beams is associated with an increased number of cracks, reduced crack spacing and reduced crack width [53]. Using this line of reasoning, a decrease in crack spacing in rubberised concrete containing BCBP herein was reasoned to have yielded reduced crack width. Concrete containing BCBP exhibited a 17.98% reduction in maximum crack space compared to control concrete. An increase in strain distribution in modified concrete resulting in an increased number of cracks with decreased width of crack could be suggested as the cause of such behaviour [35, 53].

#### 3.2.3 Ductility properties

**Figure 13:**
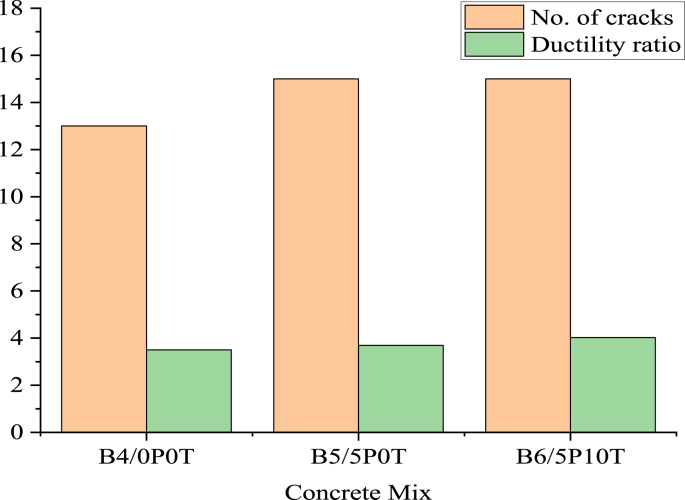
Effect of WTR content on concrete incorporated with BCBP.

The influence of WTR on a number of cracks and ductility ratio of concrete containing BCBP was investigated. The findings are illustrated in Figure 13 which demonstrates the increase in ductility ratio for rubberised concrete incorporating BCBP compared to control concrete. The increase in ductility for B6/5P10T was 14.59% compared to B4/0P0T. Findings from B5/5P0T indicated a 5.28% increase in ductility ratio in comparison with control concrete. Identical numbers of cracks were noticed for B5/5P0T and B6/5P10T which surpassed those for B4/0P0T by 2 cracks.

The reduction of load yielding failure was attributed to reduced section stiffness of B6/5P10T in comparison with the control concrete. Such phenomenon has been explained in the preceding section of beams that failed in flexure. In comparison with beams that failed in flexure, it is clear that under the same reinforcement conditions, the failure load of beams is likely to increase following positioning the loads close to the supports.

#### 3.2.4 Concrete and steel strains

**Figure 14:**
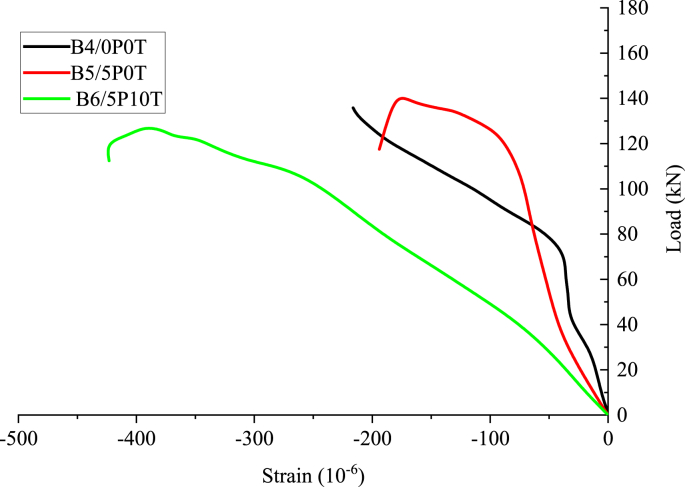
The experimental load-strain curve of concrete mixes.

Figure 14 shows the concrete strains of the tested beams plotted with reference to load. The strains were monitored using strain gauges embedded at 45° from the supports as described in Section 2. The initial increase in strains corresponding to applied load is characterised by an approximately linear behaviour. The linearity appears to be very clear for rubberised concrete incorporating BCBP compared to other concrete mixes. A discrepancy exists on the slopes of 5P0T and 5P10T and this might due to slip of the strain gauges during the recording of the strains. Straining of concrete during continued load application could be suggested as another possible reason.

The load-strain graph for rubberised concrete containing BCBP illustrates an increased negative slope attributed to its low modulus of elasticity. By comparing Figures 11 and 14, a considerable relationship exists between them. In both figures, rubberised concrete with 5% BCBP exhibits a ductile behaviour compared to the other two beams. Detection of steel strains using strain gauges attached to the tensile reinforcement steel gave incomplete data not useful for analysis. For this reason, it was decided that steel strains be neglected as no reliable analysis could have been drawn from available data. The suggested reason was the damage of the strain gauges during the loading phase. Electrical strain gauges have been observed to be unreliable or inoperative due to damage [54]. Failure of the bond between the gauges and steel resulting in slipping of the gauges could be another probable reason.

#### 3.2.5 Experimental and theoretical shear strength capacities

In this section, the shear capacities (V_pred_) for all tested beams subjected to loading were computed using BS [55] (Equation 2) and ACI [25] (Equation 3) as follows:

(2) $$Vpred\;=\;0.17ℷ\sqrt{f^{\prime }cbvd}$$

(3) $$[Vpred\;=\left[0.79\left(100\frac{As}{bvd}\right)1/3\left(\frac{100}{d}\right)1/4\left(\frac{fcu}{25}\right)1/3\right]\text{bvd}$$

where ג is the concrete density modification factor (0.75 for all lightweight concrete, 0.85 for sand-lightweight concrete and 1 for normal-weight concrete); f'_c_ is the cylinder compressive strength (MPa); f_cu_ is the cube compressive strength (MPa); A_s_ is the area of tension reinforcement (mm^2^), b_v_ is the beam width (mm) and d is the beam effective depth (mm). Because of the increased densities of all concrete mixes, the beams were not in the category of lightweight concrete. As such, the proposed interpolation expression following clause 8.6.1 of the ACI code [55] was not employed. Instead, ℷ was set to 1 for all beams and this constitutes a deficiency in the equation since it was based on conventional concrete. Further investigations are suggested in an attempt to capture the effect of tire rubber and brick powder attributes on the shear performance of concrete beams [35].

**Figure 15:**
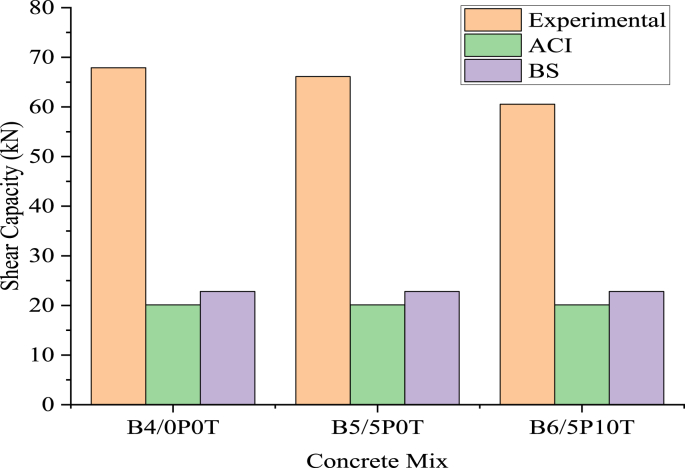
Experimental and predicted shear capacities.

**Figure 16:**
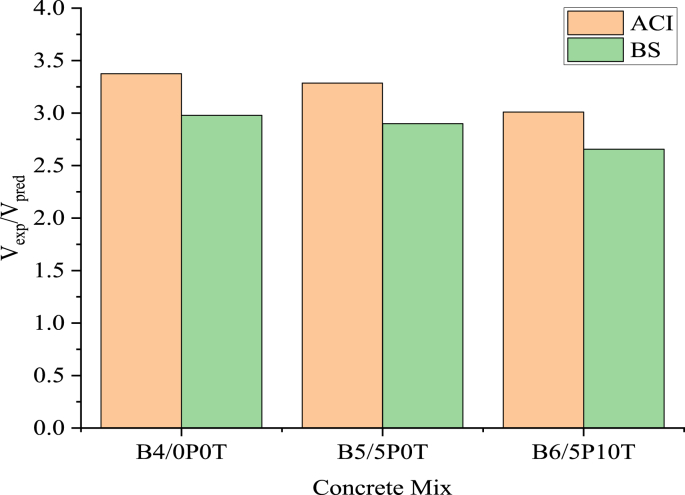
The ratio of experimental shear capacity to predicted shear capacity by ACI and BSI.

Figure 15 shows the experimental and predicted shear capacities of the tested beams. In Figure 16, experimental-to-predicted shear capacities ratios (V_exp_/V_pred_) for all beams are presented. In general, both ACI and BS enormously underestimated the shear capacity of the beams. The underestimation was more pronounced in ACI compared to BS. For both standards, the V_exp_/V_pred_ ratios were observed to decrease for B4/0P0T, B5/5P0T and B6/5P10T respectively. The ratios attained in rubberised concrete incorporated with BCBP surpassed that of control concrete by 10.82% for both ACI and BS. In another study [35], concrete beams containing 5% tire rubber and 0.35 steel fiber showed a high V_exp_/V_pred_ ratio of 2.21 using ACI.

As mentioned earlier, both ACI and BS showed limitations in the computation of predicted shear capacity values. Both standards neglect the contributions of BCBP and WTR on the shear behaviour of beams. Interpolation of concrete density modification factor in ACI is only applicable for concrete densities ranging from 0.1 kcf (1601 kg/m^3^) to 0.135 kcf (2161.35 kg/m^3^) as specified in the standard [55]. However, the inclusion of experimental values in the ratios seems to demonstrate acceptable trends among the beams.

## 4 Conclusions and recommendations

Based on the outcomes from this study, these conclusions have been reached.

1. For the beams that failed in flexure, the ductility of rubberised concrete beams containing 5% BCBP and 10% WTR improved by 23.47% compared to control concrete. This increase in ductility was evidenced with only a 15.31% reduction in flexural load. The existence of 5% BCBP in concrete demonstrated marginal improvement in ductility of 3.38% with just 2.78% load reduction compared to the control concrete.
2. The beam containing 5% BCBP and 10% WTR failing in shear and flexure exhibited 14.59% ductility improvement with 16.33% load reduction in comparison to the control concrete. Beam with 5% BCBP failing in shear and flexure gave 5.28% ductility enhancement with 7.93% load reduction compared to the control beam.
3. It is possible to achieve improved ductility without substantial loss in ultimate failure load by using 5% BCBP and 10% WTR. As such, concrete with 5% BCBP and 10% WTR has been established as a potential candidate in seismic applications.
4. Failure of rubberised concrete beams containing 5 % BCBP was dominated by branched cracks compared to normal concrete beams especially in beams that failed in flexure.
5. Numerical simulations were not combined with experimental findings in this study. The findings from this research could be used in future research to formulate numerical models. The models can be useful in the prediction of the performance of rubberised concrete incorporated with BCBP.

## Declarations

### Author contribution statement

David Sinkhonde: Conceived and designed the experiments; Performed the experiments; Analyzed and interpreted the data; Contributed reagents, materials, analysis tools or data; Wrote the paper.

Richard Ocharo Onchiri, Walter Odhiambo Oyawa & John Nyiro Mwero: Conceived and designed the experiments; Analyzed and interpreted the data.

### Funding statement

This work was supported by the 10.13039/501100015776African Union Commission.

### Data availability statement

Data will be made available on request.

### Declaration of interests statement

The authors declare no conflict of interest.

### Additional information

No additional information is available for this paper.

## References

[1] Thomas B.S., Gupta R.C., Mehra P., Kumar S. (2015). Performance of high strength rubberized concrete in aggressive environment. Construct. Build. Mater..

[2] Sana K., Paraschiv M., Kuncser R., Tazerout M. (2014). Managing the environmental hazards of waste tires. J. Eng. Des..

[3] Senin M.S. (2017). The durability of concrete containing recycled tyres as a partial replacement of fine aggregate. IOP Conf. Ser. Mater. Sci. Eng..

[4] Hall M.R., Battal K. (2014). Structural behaviour and durability of steel-reinforced structural Plain/Self-Compacting Rubberised Concrete (PRC/SCRC). Construct. Build. Mater..

[5] Key D. (1988).

[6] Zheng L., Sharon H.X., Yuan Y. (2008). Experimental investigation on dynamic properties of rubberized concrete. Construct. Build. Mater..

[7] Najim K.B., Hall M.R. (2010). A review of the fresh/hardened properties and applications for plain- (PRC) and self-compacting rubberised concrete (SCRC). Construct. Build. Mater..

[8] Fattuhi N.I., Clark L.A. (1996). Cement-based materials containing shredded scrap truck tyre rubber. Construct. Build. Mater..

[9] Duthinh D., Starnes M. (2001).

[10] Booth E. (2014). Earthquake design practice for buildings. Earthq. Des. Pract. Build..

[11] Gunasekaran K., Annadurai R., Kumar P.S. (2013). Study on reinforced lightweight coconut shell concrete beam behavior under flexure. Mater. Des..

[12] Ismail M.K., Hassan A.A.A. (2017). Ductility and cracking behavior of reinforced self-consolidating rubberized concrete beams. J. Mater. Civ. Eng..

[13] Fawzy H.M., Suzan A.A., Elshazly F.A. (2020). Properties of Rubberized concrete properties and its structural engineering applications – an overview. Egypt. Int. J. Eng. Sci. Technol..

[14] Ismail M.K., Hassan A.A.A. (2016). Applicability of using waste rubber in structural applications. Science (Wash. D C).

[15] Fantilli A.P., Chiaia B. (2018). Mechanical performances of mortar prisms and concrete slabs incorporating rubber aggregates. Mech. Res. Commun..

[16] Bektas F., Wang K. (2011). Performance of ground clay brick in ASR-affected concrete: effects on expansion , mechanical properties and ASR gel chemistry. Cement Concr. Compos..

[17] Ge Z., Wang Y., Sun R., Wu X., Guan Y. (2015). Influence of ground waste clay brick on properties of fresh and hardened concrete. Construct. Build. Mater..

[18] Bektas F., Wang K., Ceylan H. (2008). Use of ground clay brick as a pozzolanic material in concrete. J. ASTM Int. (JAI).

[19] BS EN 197-1 (2000).

[20] ASTM C618 (2003).

[21] Bhattacharjee B. (2019).

[22] ASTM C109-11 (2012).

[23] ASTM C143 (2018).

[24] BS EN 12390-5 (2009).

[25] BS 8110 (1997).

[26] BS 8666 (2000).

[27] Sargent P. (2015).

[28] Tasalloti A., Chiaro G., Murali A., Banasiak L. (2021). Physical and mechanical properties of granulated rubber mixed with granular soils—a literature review. Sustainability.

[29] Emiroglu M., Kelestemur M.H., Yildiz S. (2007). Proceedings of 8th International Fracture Conference.

[30] Ismail M.K., Hassan A.A.A. (2016). Performance of full-scale self-consolidating rubberized concrete beams in flexure. ACI Mater. J..

[31] Turatsinze A., Garros M. (2008). On the modulus of elasticity and strain capacity of Self-Compacting Concrete incorporating rubber aggregates. Resour. Conserv. Recycl..

[32] Garros M., Turatsinze A., Granju J.L. (2006).

[33] Fayyad T.M., Lees J.M. (2017). Experimental investigation of crack propagation and crack branching in lightly reinforced concrete beams using Digital Image Correlation. Eng. Fract. Mech..

[34] Vegt I., Van Breugel K. (2001). Failure mechanisms of concrete under impact loading. Fract. Mech. Concr. Concr. Struct..

[35] Ismail M.K., Hassan A.A.A. (2017). Shear behaviour of large-scale rubberized concrete beams reinforced with steel fibres. Construct. Build. Mater..

[36] Bentz E.C., Collins M.P. (2006). Development of the 2004 Canadian standards association (CSA) A23.3 shear provision for reinforced concrete. Can. J. Civ. Eng..

[37] Asiri A.M., Inamuddin M., Mohammad A. (2018).

[38] Bsisu K.A., Hunaiti Y., Younes R. (2012). Flexural ductility behavior of strengthened reinforced concrete beams using steel and CFRP plates. Jordan J. Civ. Eng..

[39] Hernández-Olivares F., Barluenga G., Bollati M., Witoszek B. (2002). Static and dynamic behaviour of recycled tyre rubber-filled concrete. Cement Concr. Res..

[40] Xue J., Shinozuka M. (2013). Rubberized concrete: a green structural material with enhanced energy-dissipation capability. Construct. Build. Mater..

[41] Fantilli A., Chiaia B. (2013). The work of fracture in the eco-mechanical performances of structural concrete. J. Adv. Concr. Technol..

[42] Li L.J., Tu G.R., Lan C., Liu F. (2016). Mechanical characterization of waste-rubber-modified recycled-aggregate concrete. J. Clean. Prod..

[43] Al-Sakini J.S. (1998).

[44] Haryanto Y., Hermanto N.I.S., Pamudji G., Wardana K.P. (2017). Compressive strength and modulus of elasticity of concrete with cubed waste tire rubbers as coarse aggregates. IOP Conf. Ser. Mater. Sci. Eng..

[45] Taylor H.P.J. (1974). The fundamental behavior of reinforced concrete beams in bending and shear. ACI Struct. J..

[46] Mousa M.I. (2015). Flexural behaviour and ductility of high strength concrete (HSC) beams with tension lap splice. Alexandria Eng. J..

[47] Shao J., Gao J., Zhao Y., Chen X. (2019). Study on the pozzolanic reaction of clay brick powder in blended cement pastes. Construct. Build. Mater..

[48] Ahmad S.H., Xie Y., Yu T. (1995). Shear ductility of reinforced lightweight concrete beams of normal strength and high strength concrete. Cement Concr. Compos..

[49] Bhattacharya S.P. (2012). Importance of ductile detailing in earthquake resistant reinforced concrete frame building. Disaster Adv..

[50] Kwan A. (2014). Ductility design of high-strength concrete beams and columns. Adv. Struct. Eng..

[51] Alasmari H.A., Bakar B.H.A., Akil H.M. (2020). AIP Conf. Proc..

[52] Barris C., Torres L., Comas J., Miàs C. (2013). Cracking and deflections in GFRP RC beams: an experimental study. Compos. Part B Eng..

[53] Alengaram U.J., Jumaat M.Z., Mahmud H. (2008). Ductility behaviour of reinforced palm kernel shell concrete beams. Eur. J. Sci. Res..

[54] Gersch B.C., Moore W.H. (1959).

[55] ACI Committee 318 (2008).

